# WHO Indicators for Valid Assessment of Iodine Deficiency Disorders

**DOI:** 10.4103/0970-0218.55303

**Published:** 2009-07

**Authors:** Umesh Kapil

**Affiliations:** Public Health Nutrition, AIIMS, New Delhi-110 029, India E-mail: umeshkapil@yahoo.com

Sir,

I read the interesting article entitled “Prevalence of Goiter in the Rural Area of Belgaum District, Karnataka,” published in the IJCM issue of January - March, 2009.(1)

The authors have assessed the goiter, by examining 950 subjects of all the age groups, in two villages, and documented the prevalence. They have utilized the World Health Organization (WHO) criteria for the classification of the grades of goiter.

WHO in 1994, had recommended indicators to assess the prevalence of Iodine Deficiency Disorders (IDD) in a community.(2) The age group recommended for IDD survey (goiter survey) was 6 - 12 years. This age group was recommended to be used in the surveys in all the countries because children in this age group have a high vulnerability, easy access, and are useful for surveillance of other health activities. Also, affected children develop an enlarged thyroid, in response to iodine deficiency and can be readily examined in large numbers in school settings.

Earlier, the scientists in different countries / regions used various age groups for assessment of the prevalence of goiter (iodine deficiency). This was leading to a collection of data, which was from heterogeneous age groups. WHO brought out the publication on IDD indicators(2) so that scientists could use uniform guidelines for the collection of data on goiter prevalence.

The WHO guidelines are presently being used globally and children of 6 to12 years are included. Use of a uniform age group helps in comparison of research findings of the various surveys conducted by the scientists in different regions and nations. National and International data bases are being developed for comparison using such data.

The goiter in adults is usually of a long duration, and does not disappear after the implementation of the Universal Iodine Supplementation Program, as fibrous bands develop in the enlarged thyroid gland. When the results of a repeat goiter survey, include adult population, they provide incorrect data on the effectiveness of the iodine supplementation program, as adult goiters may remain nonresponsive to iodine supplementation.

Authors in their publications,(1) have discussed and compared the prevalence of goiter found in their studies with other earlier studies, which were conducted in different age groups such as, 8 to 10 years, 6 to 15 years, 13 to 18 years, and so on. Such comparisons have limitations as they are not scientifically valid. We must follow the simple principle of comparison according to which “like should be compared with like,” that is, “apples with apples” and “oranges with oranges” and not vice versa.

WHO has published a series of books on the Global Status of Micronutrient Deficiency in different countries in the world.(3) One such book documents status of iodine deficiency. This book compiles the data of all published studies, in indexed journals, on prevalence of goiter, according to the age group.
